# A Deep Learning Approach for Fusing Sensor Data from Screw Compressors

**DOI:** 10.3390/s19132868

**Published:** 2019-06-28

**Authors:** Serafín Alonso, Daniel Pérez, Antonio Morán, Juan José Fuertes, Ignacio Díaz, Manuel Domínguez

**Affiliations:** 1 Grupo de Investigación en Supervisión, Control y Automatización de Procesos Industriales (SUPPRESS), Esc. de Ing. Industrial, Informática y Aeroespacial, Universidad de León, Campus de Vegazana s/n, 24007 León, Spain; 2 Electrical Engineering Department, University of Oviedo, Edif. Departmental 2, Campus de Viesques s/n, 33204 Gijón, Spain

**Keywords:** sensor data fusion, deep learning, convolutional neural networks, screw compressors, capacity control system

## Abstract

Chillers are commonly used for thermal regulation to maintain indoor comfort in medium and large buildings. However, inefficiencies in this process produce significant losses, and optimization tasks are limited because of accessibility to the system. Data analysis techniques transform measurements coming from several sensors into useful information. Recent deep learning approaches have achieved excellent results in many applications. These techniques can be used for computing new data representations that provide comprehensive information from the device. This allows real-time monitoring, where information can be checked with current working operation to detect any type of anomaly in the process. In this work, a model based on a 1D convolutional neural network is proposed for fusing data in order to predict four different control stages of a screw compressor in a chiller. The evaluation of the method was performed using real data from a chiller in a hospital building. Results show a satisfactory performance and acceptable training time in comparison with other recent methods. In addition, the model is capable of predicting control states of other screw compressors different than the one used in the training. Furthermore, two failure cases are simulated, providing an early alarm detection when a continuous wrong classification is performed by the model.

## 1. Introduction

Nowadays, tendencies in industry are leading to more interconnected equipment due to the development of the Internet of Things (IoT) and cyber-physical systems in the so-called Industry 4.0 [1]. This provides advantages in terms of information from sensors and additional systems that can help to acquire domain knowledge about complex processes. The incorporation of large amounts of sensors will be a requirement in future industrial installations. However, the transformation of current industrial facilities that are working perfectly is slower in order to be more competitive in a secure way.

Heating, ventilation, and air conditioning (HVAC) systems usually provide the regulation of thermal comfort for any common indoor environment. Due to their size, medium-sized and large facilities require centralized systems that manage a group of chillers [2] included in a chiller plant in order to remove heat using different methods, e.g., air-cooled or water-cooled chilling. Apart from the evaporator, the condenser, expansion valve, and other elements, a chiller includes several compressors of the same type (reciprocating, screw, scroll compressors, etc.) in order to accomplish the task. Obviously, these machines play an essential part in the energy efficiency and consumption demand of the whole facility [3]. Therefore, any optimization in the efficiency of control systems or fault detection entails a great enhancement in facility operation [4]. Nevertheless, the possibility of further studies of these machines, e.g., with respect to efficiency or maintenance of the compressor, is complicated because of difficult accessibility, in general caused by their own design (many of them are semi-hermetic) or limitations imposed by the manufacturer. Moreover, it would require the installation of more sensors, which is not straightforward for several reasons such as physical viability, functional structure of the plant, or additional costs.

Data integration from multiple sources has been developed [5] to produce more comprehensive information that provides not only more reliable data than from an individual source but also, in some cases, unique information that would be otherwise inaccessible. In this way, data-based algorithms can address the challenge of fusing data from a complex system to obtain effective information. Recent advances in deep learning approaches, based on neural networks, have demonstrated promising results in several applications [6] such as computer vision or speech recognition. These neural networks have proven their ability to learn new representations of data at different levels of abstraction, which matches perfectly with the data fusion problem. If the result of data fusion provides information about the process, this can be transformed into knowledge to support decisions about the process by means of tasks such as monitoring of working conditions and fault detection.

In this work, a deep learning approach is proposed for data fusion of several variables from a screw compressor to predict control states and perform real-time monitoring so that great differences from the model detect possible sources of failures that would require further analysis. The main contributions of this work are the creation of a data fusion model based on time-series data analysis techniques and deep neural networks to estimate current control states of a screw compressor in a chiller, the comparison of this model with others from the state-of-the-art in order to select an effective one, the scalability of the approach evaluating the performance on different compressors, the behavior in terms of early fault detection using simulated situations of failure, and last but not least, validation is undertaken using real data on screw compressors from a large air-cooled chiller located at the plant of the Hospital of León.

This paper is organized as follows. Related work is reviewed in Section 2. Section 3 states the problem. In Section 4, the proposed methodology is exposed. Here, the approach based on deep learning is explained in detail. In Section 5, the experiments are presented and results are discussed. Finally, conclusions are drawn in Section 6.

## 2. Related Work

Data fusion is a multidisciplinary research field that uses techniques from different areas [5,7,8] such as artificial intelligence or information theory. Despite the number of different definitions for multi-sensor data fusion existing in the literature [9], most of them share similar characteristics. First of all, the transformation of information includes possible methods for combining data, which can usually come from multiple and heterogeneous sources. The way this transformation is performed, e.g., automatically or semi-automatically, is another aspect to take into account, because the results of fusion should facilitate effective decision-making situations for humans.

Generally, the main benefits of sensor fusion not only include the increase of accuracy, but also an improvement in the reliability of data. However, multi-sensor data fusion deals with several challenging issues. For instance, signal acquisition from multiple sensors may cause problems that make feature extraction more difficult than using a single sensor. These can cause noise and uncertainties that corrupt data information. A wide variety of data fusion methods are reviewed in [8], where existing algorithms are explored according to these data-related aspects. The application of these algorithms is growing to many potential fields such as sensor networks or image processing, which motivates more active data fusion research. Data fusion was already applied to building central chiller plants [10] with the aim of increasing the accuracy of building cooling load measurement through the use of a combination of direct and indirect measurements. Direct measurement is computed based on differences in the temperature and flow rate of chilled water, whereas indirect measurement is based on evaporating/condensing temperature and electrical power input. A merged measurement is constructed when direct measurement is corrupted by noise or when systematic errors and outliers are detected, so that a certain confidence degree is achieved.

In addition, data-based algorithms have been used for fault diagnosis in chiller systems. For example, Beghi et al. [11] proposed a method based on principal component analysis to distinguish anomalies from normal operation and an isolation of the variables related to faults using experimental data from a centrifugal chiller system. Moreover, fault diagnosis solutions combine several methods such as statistical modeling, machine learning, or classical data fusion techniques in order to achieve stable detection rates. For instance, an online hybrid method is proposed in [12], combining an extended Kalman filter and recursive one-class support vector machine (ROCSVM) to detect typical chiller faults. The method is trained only using normal data and transition data samples from normal to faulty are neglected. Results show an enhanced detection accuracy with respect to others using a previous feature selection.

Many previous works have addressed the challenge of accurately classifying time series data. A comparison of recent proposed algorithms is carried out in [13]. However, a practical deployment of these solutions in a real-time system is still computationally limited. Recently, deep learning models [6] have achieved remarkable results in classification tasks for several applications such as computer vision [14], image classification [15], and natural language processing [16]. Similarly, a revision of recent deep learning classifiers for time series, separated into the two categories of generative and discriminative models, is developed in [17], where several approaches based on popular architectures such as multilayer perceptrons or convolutional neural networks are evaluated with regard to time series data.

Data fusion in combination with a large number of interconnected objects, which nowadays is a real option because of the Internet of Things (IoT), provides a promising future challenge in numerous application areas. A number of surveys exist. For instance, related literature is reviewed in [18], focusing on mathematical models and IoT environments, and personal sensing, i.e., studies of data from everyday devices such as smartphones or wearables to identify human information, is reviewed in [19]. More related examples are a cooperative localization algorithm for mobile sensor networks [20], a diffusion particle filtering strategy, proposed in [21] to estimate the state of non-linear systems, and an accelerator of deep learning models in mobile devices [22].

Convolutional neural networks were used with sensor fusion at the data level in [23], improving the performance of fault diagnosis for rotating machinery. An adaptive multi-sensor data fusion method is also proposed in [24] for fault diagnosis of a planetary gearbox, where the best accuracy is achieved using a deep convolutional neural network for several fusion levels. Moreover, deep-learning-based models were developed for building the cooling load prediction [25] of 24-hour profiles and compared with other traditional methods, showing an improvement of the performance when using features extracted by unsupervised deep learning techniques. Although these techniques have demonstrated remarkable results in various applications, there is still a need to study both the levels of abstraction of extraction features and the development of potential models that might allow a better estimation of control machine states in order to predict future faults effectively.

In this work, the control state of a screw compressor is predicted from fused sensor data in order to monitor functionality and detect possible failures.

## 3. Problem Statement

### 3.1. Capacity Control in Screw Compressors

Basically, screw compressors use either a four-step or continuous capacity control system. In a four-step control, four valves and their corresponding solenoids are used to open the slide valve at fixed values (25%, 50%, 75% or 100%). In contrast, in a continuous (stepless) control, the slide valve can be opened at any value using only two valves (one for increasing the capacity and the other for decreasing it). Thus, the screw compressor can displace any amount of refrigerant gas between 0 and 100%, achieving a better fit to the cooling demand. Moreover, this capacity control system is more efficient and accurate, so it is commonly implemented in the screw compressors.

In a continuous capacity control system, the full load amperage (FLA) [26], i.e., the amperes which the compressor consumes at full load (nominal displacement of the refrigeration gas), usually play an important role. This variable depends mainly on the temperatures and pressures of the refrigeration gas in the discharge and suction lines. The higher the temperatures and pressures are, the higher the compressor work is. For example, outdoor temperature provokes an increase in the condensing temperatures and pressures and, as a consequence, of the compressor consumption. Therefore, the control board continuously computes the FLA (from the difference between the current and design measurements of the temperature and pressure sensors), which is different for each situation.

(1) $$\%FLA=\frac{Amp}{FLA}*100$$

**Figure 1:**
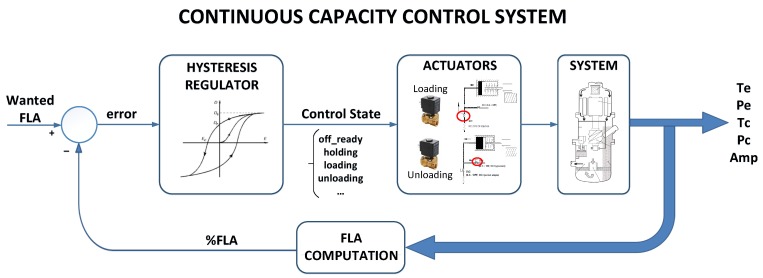
The continuous capacity control system in screw compressors using FLA (Full Load Amperage).

The percentage of FLA (%FLA), i.e., the amperes that the compressor should consume at a certain load, displacing an specific amount of refrigeration gas (see Equation (1)), is the controlled variable. The setpoint (wanted %FLA) is modified by the control board to keep the output water temperature within the specified range. Typically, an on-off (relay) regulator with hysteresis is implemented in order to keep the %FLA within the control band (see Figure 1).

As mentioned above, only two on–off valves with the corresponding solenoids are used in a continuous capacity control system: one of them for loading the compressor and the other for unloading. This is usually performed by sending pulses to these solenoids. For that purpose, the control system has to establish an appropriate control state (holding, loading, unloading, etc.), according to the FLA error (wanted %FLA–%FLA). Manufacturers define several control states for screw compressors in a normal operation (listed in Table 1). When the control board establishes a 2-Loading/3-Unloading control state, the loading/unloading solenoid is pulsed during a short period of time. While this state is set, the loading/unloading solenoid keeps receiving consecutive pulses with a certain delay between them. In a holding state, none of the solenoids will be pulsed. It should be remarked that control boards developed by chiller manufacturers are usually closed, so they do not generally allow users access to internal control variables. It is only possible with specific configuration software provided by manufacturers.

Due to the high number of operations (pulses) on loading/unloading solenoids and valves, they become a main source of faults, causing malfunctions and inefficiencies in the capacity control of the screw compressors. For instance, the control board can set the 2-Loading control state, but the capacity of the screw compressor will not increase whenever the corresponding solenoid or valve is damaged, letting the system remain in the 2-Loading control state, and the screw compressor cannot achieve the cooling demand.

Thus, a system able to detect, in real time, faults on loading and unloading solenoids or valves would be very useful. We hypothesize here that sensor data from refrigeration circuits contain information about the current control state. Fusing these sensor data and comparing them with the control state at the main board can allow us to identify the mentioned faults.

### 3.2. An Air-Cooled Chiller at the Hospital of León Comprising 3 Screw Compressors

The chiller plant at the Hospital of León consists of five air-cooled chillers (model Petra APSa 400-3) and two water-cooled chillers (model Trane CVGF 650). In this work, an air-cooled chiller with three compressors is used for the experimentation.

**Figure 2:**
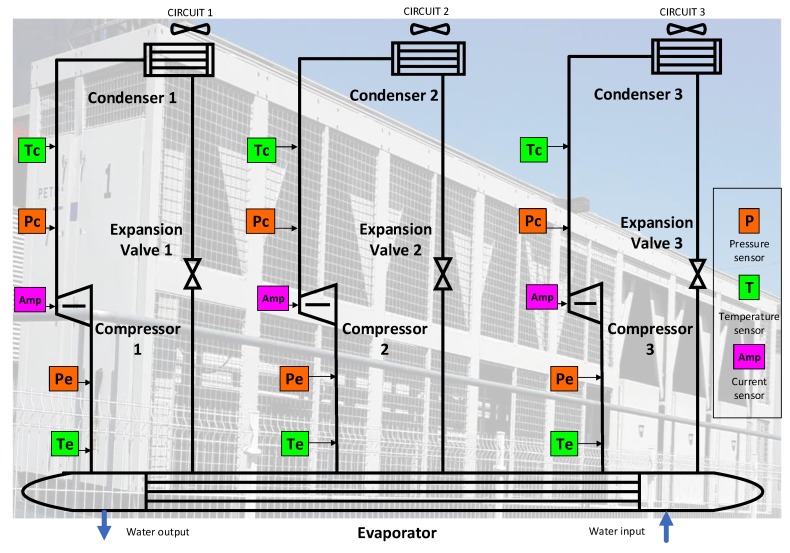
The air-cooled chiller with 3 screw compressors (real photo in the background).

The air-cooled chillers have a maximum cooling capacity of 400 tons (approximately 1407 kW) and include three refrigeration circuits. Each circuit has a typical structure with a condenser in V form, an electronic expansion valve (EEV), and a screw compressor. All of the circuits have a common evaporator (see Figure 2). These elements define three closed refrigeration cycles. The screw compressor is the RC2-790A model by Hanbell, developed especially for applications in air-conditioning and refrigeration. It allows a maximum displacement of 791 m${}^{3}$/h of R134a refrigeration gas, and it is driven by a three-phase induction motor (400 V; 109 kW). The FLA is 284A in nominal conditions (i.e., condensing temperature at 40 ${}^{\circ }$C and evaporating temperature at 0 ${}^{\circ }$C). Its capacity can be regulated between 50–100% of its maximum value by means of loading and unloading valves. Note that a lower limit (50%) is configured in order to increase the compressor’s efficiency.

Several sensors (listed in Table 2) provide information on the compressor to the control system. Most of them are located on the pipes of refrigeration gas, connecting the compressor with the evaporator or the condenser, since this screw compressor is semi-hermetic, hindering their internal installation. Furthermore, these sensors are also used for controlling the other elements of the refrigeration circuits (the evaporator, the condenser, or the expansion valve). For example, the condenser tries to maintain the condensing pressure and temperature within the design range, with independence of the outdoor temperature, air flow, etc. In the same way, the aim of the evaporator is to keep the evaporating pressure and temperature in nominal values. Therefore, the operation of these elements (evaporator, condenser, or expansion valve) directly influences the compressor since the refrigeration cycle is closed. The control board acquires the sensor data, computes the FLA and %FLA, and regulates the compressors according to the adequate control state.

**Figure 3:**
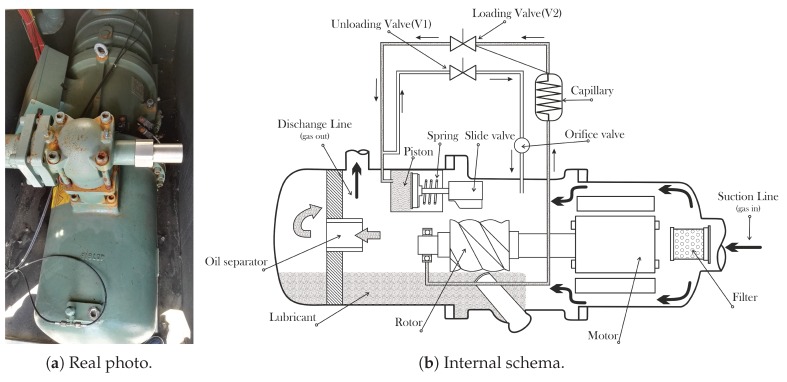
A screw compressor of the air-cooled chillers.

Figure 3 shows a picture and the internal schema of one of these three compressors. The capacity control system and other internal elements of the compressor can also be seen in Figure 3b. The capacity control system comprises a slide valve, which is driven by a piston. The oil pressure moves the piston in the cylinder. The oil flows through the filter cartridge and capillary, then fills the cylinder (the pressure is higher than the spring plus the discharge pressure gas). This causes the piston to move towards the right side in the cylinder (the slide valve moves towards the right side), so that the effective compression volume and the displacement of refrigerant gas increase. In contrast, if the high-pressure oil in the cylinder bypasses to the suction port, the piston and the slide valve move towards the left side, bypassing some of the refrigerant gas. As a result, the refrigeration capacity decreases because of the reduction of effective compression volume and the displacement of refrigerant gas. A spring is used to push the piston back to its original position.

In a continuous capacity control system, solenoid valve V2 is energized for loading and solenoid valve V1 for unloading. When V2 is energized and V1 is not, oil is injected continuously into the piston cylinder and does not bypass through V1, so the compressor keeps loading. In contrast, when V1 is activated but V2 is not, oil inside the piston cylinder is bypassed to the suction port through V1. When both V2 and V1 are not energized, the piston is held in its position. These two valves are controlled by pulsing the on–off solenoids, according to the FLA control loop. In this way, refrigeration capacity is modulated.

The main control board manages the three compressors in order to keep the chilled water temperature in range. For a smooth modulation, there is a capillary in the oil loading line and an additional orifice valve in the oil unloading line in order to avoid too fast operations for loading and unloading. It is very important to control these operations in a stable and efficient way.

## 4. A Deep Learning Approach for Fusing Sensor Data

**Figure 4:**
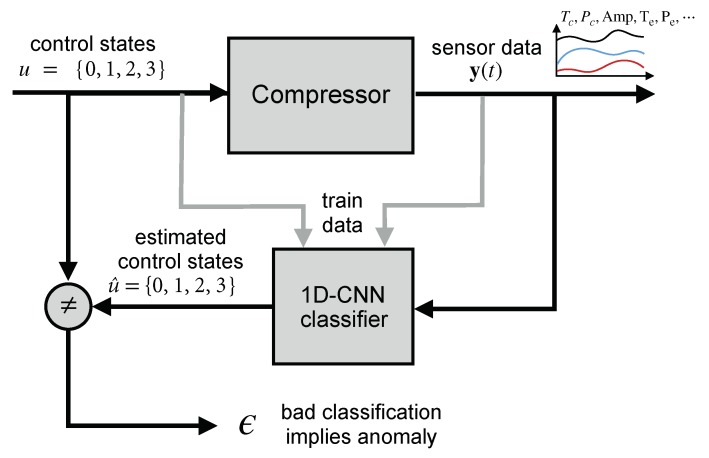
Methodology for verifying the operation of screw compressors based on a 1D CNN (one-Dimensional Convolutional Neural Network) classifier.

We propose a methodology for verifying the correct operation of the screw compressors and detecting faults in their capacity control system. Our approach estimates the control states (1-Holding, 2-Loading, 3-Unloading, etc.) using real sensor data from the screw compressors. These estimations are compared with the true control states (provided by the main control board), aiming at detecting anomalies (mainly electrical faults on loading and unloading solenoids). A classifier is used to obtain the predicted control states. It is based on a one-dimensional convolutional neural network (1D CNN). A schema of the method is shown in Figure 4.

This method considers the sensors as channels, enabling the implicit fusion of sensor data from several subsystems. The refrigeration process consists of four main subsystems (evaporator, compressor, condenser, and expansion valve) in a closed cycle. Despite that these subsystems have different natures, they are strongly coupled (the operation of a subsystem influences the remaining ones), resulting in an overall dynamical system. Sensor fusion allows obtaining more information than analyzing the sensor data of each subsystem individually. Therefore, in order to consider the overall system with interactions among four refrigeration processes, sensor data from several subsystems (compressor, evaporator, and condenser) are fused in our approach. It is based on one-dimensional convolutional neural network (1D CNN). Furthermore, the proposed fault diagnosis consists of fusion at a decision level comparing real control stages of the system with control predictions as additional information to confirm the correct performance of the system.

Data from non-faulty sensors are used to train the classifier. Once the classifier is trained, predicted control states can be obtained directly from the merged information from sensors. Our approach tackles a sensor data fusion together with a multi-classification problem. Its explanation is divided into two parts: the data level and the algorithm level.

### 4.1. Data-Level Phase

Time series from sensors usually require being reframed into a new data representation for the application of the algorithm [27]. Most of the algorithms use a fixed-length vector as the input. For that reason, a sliding overlapping window method is commonly used [28]. However, in some applications, the patterns to be identified might have different lengths due to signal dynamics, so fixed-length methods, such as the simple application of sliding windows, are bound to fail. In these cases, the window size should be dynamically adjusted by using signal information in order to determine the most effective segmentation [29].

**Figure 5:**
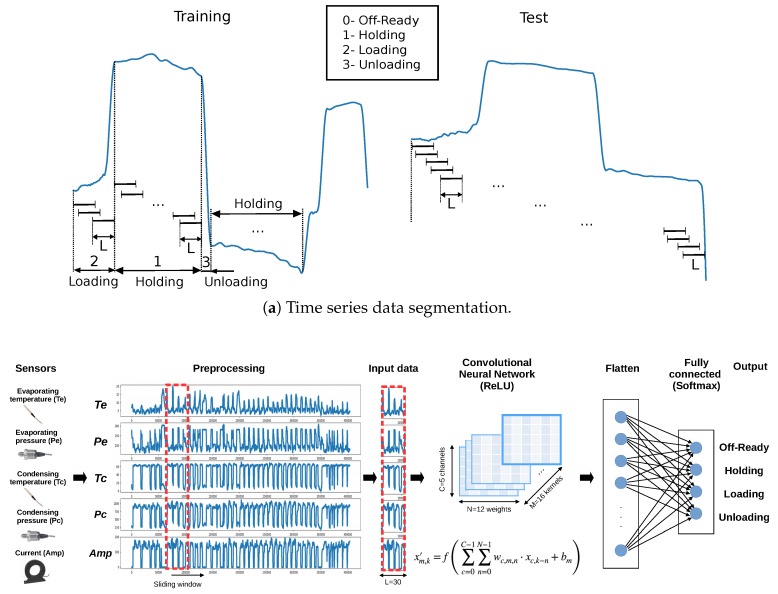
Data-level and algorithmic phases of the proposed approach.

For that reason, in this work, we propose to use a variant of the sliding window method that considers the dynamics of the compressor control. We compute fixed-length overlapping windows (*L* being the length) within the same control state in the training process, so data are augmented (see Figure 5a, left). Note that the length *L* must be smaller or equal to any of the considered periods.

The compressor can stay in the same control state during a short or a long period, depending on the cooling demand and the surrounding conditions. For example, if the cooling demand is stable, the compressor will run in holding state during a long time. On the contrary, if the cooling demand changes continuously, the holding state will last only a short time. Within the same control state, the evolution of compressor variables can be different since they depend on the outdoor temperature, the condensing air flow, input and output water temperatures, water flow, etc. In the same way, 2-Loading/3-Unloading control states can last differently, depending on the setpoint step and the surrounding conditions. Thus, the model should consider several dynamics of the compressor. In the test process, we only used basic fixed-length windows since it is considered a real-time operation (see Figure 5a, right).

### 4.2. Algorithmic Phase

We propose a one-dimensional convolutional neural network (1D CNN) to merge data from several sensors. A 1D convolutional layer is used as an input to our model in order to reduce the number of network connections and to process all information together [30]. This layer receives an input data array with a shape of *C* channels and a sliding window length of *L* time steps. 1D CNN applies a bank of *M* kernels (filters) per channel, having *N* coefficients each. The math operation that includes one bias term $b_{m}$ per kernel is

(2) $$\begin{array}{c} x_{m,k}^{\prime }=f\left(\sum_{c=0}^{C-1}\sum_{n=0}^{N-1}w_{c,m,n}\cdot x_{c,k-n}+b_{m}\right). \end{array}$$

This results in a total of $M\cdot \left((C\cdot N)+1\right)$ weights that need to be adapted, i.e., $w_{c,m,n}$ and $b_{m}$. This architecture allows us to exploit the spatial invariances [31] in the time series of the compressor variables, each kernel having to learn local motifs that can appear at different locations of its channel along the window length. It helps in achieving invariance to translations [32]. Upon training, kernels are adapted to suit relevant time motifs so that the convolution operation results in large features when these motifs are present. The results of the convolutions done on each channel are averaged. Note that despite that averaging could seem to miss the relative relevance of one channel above others, the amplitude of the kernel weights for this channel can be learned to favor it over the other channels in the aggregated result. The overall outcome of the 1D CNN is a tensor with *L* time steps and *M* filters. In Figure 5b, the proposed architecture of the network for sensor data fusion and classification is shown. It consists of 4 layers: an input layer with a dimension of (L=30,C=5), with the channels’ evaporating pressure being Pe and temperature Te, condensing pressure Pc, temperature Tc, and current at the compressor Amp; a 1D CNN layer with M=16 filters, filter length of N=12, a rectified linear unit (ReLU) activation function (in this layer, timesteps are convolved, and then channels are averaged); a flattened layer; and finally, an output layer that is a fully connected (dense) layer with 4 neurons (classes) and a softmax activation function.

## 5. Results and Discussion

Sensor data from an air-cooled chiller at the Hospital of León were collected during the winter of 2019. The chiller plant management commanded the chiller under study from approximately mid-February to mid-April. Data from sensors were stored in the corresponding logs of the building management system (BMS). The main control board of the chiller communicates with the BMS using the Modbus RTU protocol. The sampling ratio was 10 s, so the total number of samples was over 400,000 for each compressor. These data were pre-processed, and so duplicated data were dropped, timestamps of different logs were synchronized, missing values were filled with the previous known value, and finally, data were filtered using a moving average that attenuated the sensors’ noise. The number of sensors considered was 5 (evaporating pressure Pe and temperature Te, condensing pressure Pc and temperature Tc, and current at the compressor Amp).

We selected sensor data from compressor 3 since it ran longer than the other two compressors (compressors 1 and 2) during the aforementioned period. Data from compressor 3 were split into two sets: training (70%) and validation (30%). Additionally, we used all data from compressor 1 and 2 to test the scalability of our approach. Then, data were normalized between 0 and 1.

The training set was prepared as explained in Section 4.1. For that purpose, we computed fixed-length sliding windows within the same control state of the compressor. The length size *L* was set to 30 time steps, i.e., 5 min, which is lower than the minimum time of all control states (44 time steps). Therefore, faster changes on the capacity control system were not considered. Note that the building cooling load was expected to have slow dynamics. The validation set was computed using a continuous fixed-sliding window (L=30) for a real-time operation. Thus, the training patterns were approximately 267,000, whereas the validation patterns were 120,000. On the other hand, test sets from compressor 1 and 2 contained about 400,000 and 320,000 samples, respectively, slightly lower than compressor 3.

The categorical classes (0-Off-ready (25%), 1-Holding (48%), 2-Loading (16%), and 3-Unloading (11%)) were converted using the one-hot encoding method. As indicated, classes are rather imbalanced. Holding is the most common control state since the cooling load in the building does not vary considerably and has slow dynamics.

Considering that the datasets are imbalanced, we report average performances, such as the micro-average (averaging the total true positives, false negatives, and false positives) and the weighted macro-average (averaging the support-weighted mean per class). Furthermore, the selected metric to evaluate the approach is the F1-score [33,34].

Additionally, we used the confusion matrices, which give an overall glance at the multi-classification problem, and prediction–recall curves (PR curves). Unlike the well-known receiver operating characteristic curves (ROC curves) (very popular for evaluating a classification problem), PR curves are not impacted by imbalanced datasets [35]. Other alternatives can be applied to deal with imbalanced data, such as the synthetic minority over-sampling technique (SMOTE) [36] or the k-Fold technique [37].

### 5.1. Fusing Sensors with 1D CNN

The model consists of a 1D CNN layer whose input shape is (30, 5). The non-linear activation function is a rectified linear unit (ReLU). The number of filters is 16 in order to capture all the different dynamics within the same control state. After flattening data, the output layer has 4 classes and the activation function is softmax (a probabilistic distribution function of belonging to each class). After several runs, the filter length was set to 12 time steps. The training epochs were set to 20 and the Adam optimizer [38] was used to minimize a categorical cross-entropy loss function and maximize the accuracy.

**Figure 6:**
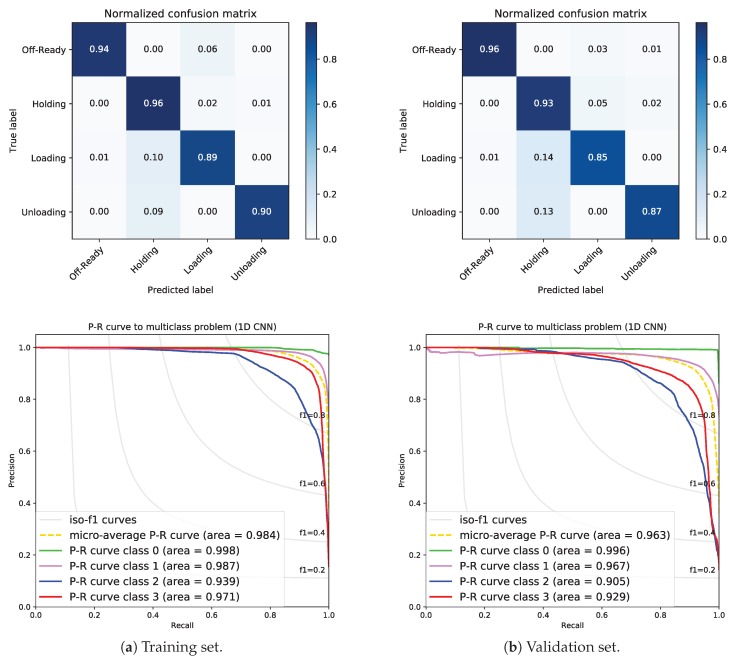
Confusion matrices and prediction–recall curves (PR) curves using sensor data from compressor 3.

Here, we present the performance of our approach (1D CNN) for fusing sensor data from screw compressors. Figure 6 shows the results (confusion matrices and PR curves) for training (see Figure 6a) and validation (see Figure 6b) datasets with data from compressor 3. A priori, the results are encouraging. As expected, classes 0-Off-Ready and 1-Holding are easily detected (>90%), whereas the classifier performs worse with classes 2-Loading and 3-Unloading (≤90%), even though the main aim is specifically to detect 2 and 3 control states with accuracy. Focusing on class 2-Loading, we observe that sometimes (10% in training and 14% in validation) it is wrongly classified. The same happens for class 3-Unloading (9% in training and 13% in validation). These incorrect classifications were analyzed, revealing that they just occur at some transition (change) points of the control state. The longer the sliding window is, the worse the performance will be, i.e., the detection will be more delayed.

The area under the PR curve (AUC) for each class is also included in Figure 6.

Moreover, micro- and weighted macro-F1-scores for training and validation datasets from compressor 3 are listed in Table 3. Both are high (>90%).

### 5.2. Comparison with Other Fusing Methods

**Figure 7:**
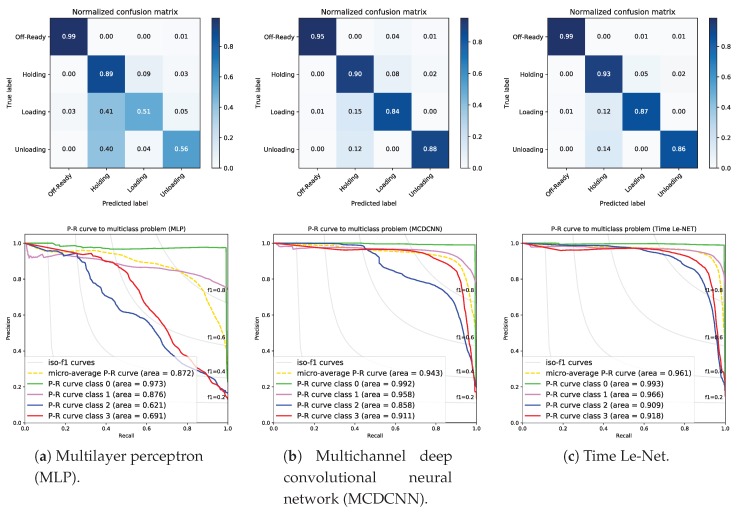
Comparison of the confusion matrices and PR curves using sensor data from compressor 3 (validation set).

The proposed approach is compared with other state-of-the-art methods useful for fusing sensor data. The methods reviewed in [17] were tested, and here, results from the most relevant ones are presented, including multilayer perceptron (MLP), multichannel deep convolutional neural network (MCDCNN), and time Le-Net. Results can be observed in Figure 7.

The MLP model consisted of 5 layers, an input layer with a dimension of 5, 3 hidden layers (500 units each), and an output layer (4 units). A dropout regularization of 0.1–0.2 was used between layers to avoid overfitting, and ReLU was selected as an activation function, except in the output layer (softmax). It was trained with a backpropagation algorithm.

The MCDCNN model implemented five independent 1D convolutions (in parallel) on each channel, i.e., on each sensor. For each channel, it consisted of 6 layers. The input layer had a dimension of (30,1). Then, two identical 1D CNN with 8 filters, filter length of 5, and a ReLU activation function were followed by two max poolings of 2 and a flatten layer. Finally, all channels were concatenated to a dense layer with 732 units and ReLU activation function. The output layer had 4 units and a softmax activation function. The resulting network architecture is more complex.

The time Le-Net model consisted of 7 layers, an input layer with a dimension of (30,5), a first 1D CNN with 5 filters, a filter length of 5, and a ReLU activation function. Then, a max pooling of 2 was performed. The second 1D CNN had 20 filters, a filter length of 5, a ReLU activation function. Then, a new max pooling of 4 was performed, to flatten data. Finally, a dense layer with 500 units and ReLU activation function was implemented. The output layer had 4 units and a softmax activation function. As you can see, it has a more complex network architecture, including two 1D convolutions.

For all methods, the training epochs were 20—likewise with the 1D CNN. An Adam optimizer was also used to minimize a categorical cross-entropy loss function and maximize the accuracy.

As expected, MLP has a poor performance (see Figure 7a), specifically on class 2-Loading and 3-Unloading, since it only merges the current measurements of the 5 sensors. In contrast, 1D CNN, MCDCNN, and time Le-Net fuse the *L* time steps (current and previous measurements) of the 5 sensors. MCDCNN has an acceptable performance (see Figure 7b), except on class 2-Loading. The main different between MCDCNN and our approach is the way of performing the convolutions. MCDCNN implements 5 independent 1D convolutions (in parallel) on each channel and then it concatenates them, whereas our approach performs a 1D convolution, averaging all sensors. Time Le-Net has an outstanding performance (see Figure 7c) similar to the 1D CNN performance, but it implements a more complex network architecture, including two 1D convolutions and two max pooling layers. Comparing the F1-score (micro- and weighted macro-; see Table 4), we can ascertain that 1D CNN and time Le-Net are two excellent methods for fusing sensor data and have a similar performance (better than MLP and MCDCNN), but time Le-Net needs more time for training. Furthermore, the 1D CNN method is able to predict a sample 90 milliseconds faster than time Le-Net, providing a certain advantage for real-time monitoring.

Summarizing, our approach based on 1D CNN allows us to merge sensor data, providing a high accuracy and consuming less time for training, with a simpler network architecture.

### 5.3. Generalization to Other Screw Compressors

In order to demonstrate the scalability of our approach to other screw compressors, we tested the 1D CNN model built with sensor data from compressor 3 using data from the other two compressors (1 and 2). We consider that the 3 screw compressors have identical internal parts, so they work in a similar way. However, some deviations could exist due to the number of running hours, new elements in a reparation, oil levels, the amount of refrigeration gas, etc.

**Figure 8:**
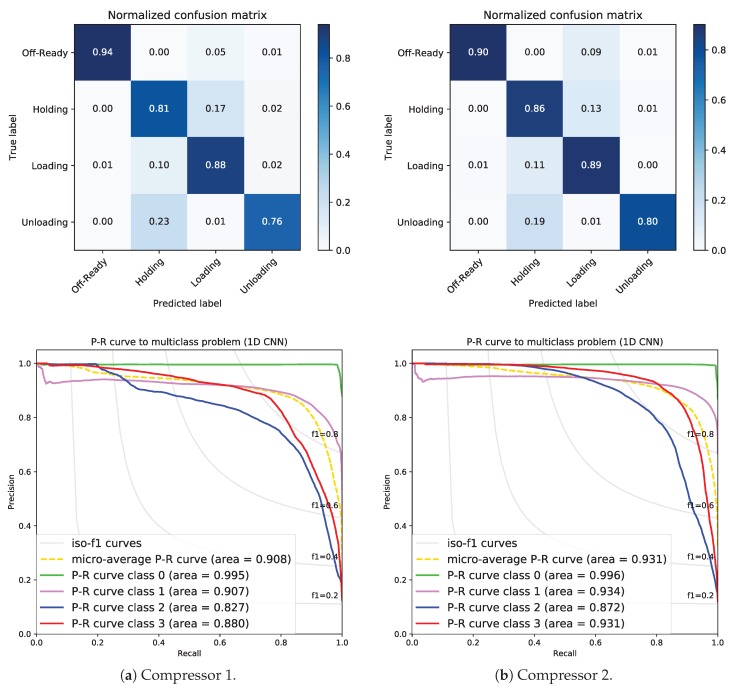
Confusion matrices and PR curves using sensor data from similar compressors 1 and 2.

Compressor 2 was running a similar time, but compressor 1 was running for less time than compressor 3 and 2 due to a start failure. We used the whole dataset (100%) from compressors 1 and 2 for testing. Figure 8 shows the results. As you can see in Figure 8a, the performance of our approach using the test dataset from compressor 1 is not very good. There are a few control states that are erroneously classified. For example, 17% of class 1-Holding is wrongly classified as 2-Loading. Furthermore, 23% of class 3-Unloading is wrongly classified as 1-Holding.

The performance of our approach using the test dataset from compressor 2 is slightly better than from compressor 1 (see Figure 8b). Nevertheless, 19% of class 3-Unloading is wrongly classified as 1-Holding.

To sum up, sensor data from several compressors (not only from one) should be captured in order to be used to train a model, so that it contains more information about the operation of the capacity control system.

Micro- and weighted macro-F1-scores with test datasets from compressor 1 and 2 are listed in Table 5. In this case, both are not so high (<90%).

### 5.4. Detecting Faults on Valves or Solenoids

Finally, we demonstrate the use of our approach for detecting anomalies in the loading and unloading valves or solenoids. This can provide a huge help to the maintenance staff in order to identify a problem that worsens the compressor efficiency and hinders fitting the cooling load. In on monitoring, the proposed 1D CNN classifier predicts the control state based on fusing sensor data from the compressor (*L* samples each), and then it is compared with the real control state. If both control states (real and predicted) are different, it indicates an anomaly, i.e., these consequences (sensor data) should not be caused by this control state.

The datasets do not contain faulty data, so we simulated the anomalies to test the detection of problems in loading and unloading valves or solenoids. For that, we simulated faulty data containing two typical anomalies on loading and unloading solenoids (one each). The typical fault is provoked by pulsing the solenoids many consecutive times. They get hot and end up burning. In both cases, an incorrect operation of the compressor capacity is simulated. When control states command actions like loading (or unloading), the compressor should increase (or decrease) its capacity. However, it will keep stable (similar to running in a holding control state) due to the failure. Therefore, the faulty sensor data are generated assuming a steady behavior of the variables (pressures, temperatures, and current consumption).

**Figure 9:**
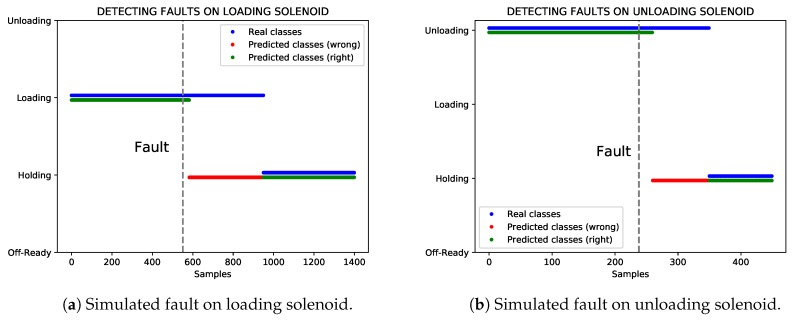
Detecting faults on loading and unloading solenoids.

In Figure 9, we can see the results of the simulated data and make out the use of our approach for detecting faults. Figure 9a represents the detection of faults on the loading solenoid, whereas Figure 9b shows the detection of faults on the unloading solenoid. The dashed vertical line indicates when the fault occurs in both cases. Real control states (blue) and predicted control states using the 1D CNN model built with sensor data from compressor 3 are represented. The 1D CNN classifier correctly predicts the control states (green), except when the fault appears (red). Note that the classifier indicates faults with a short delay (normally <5 min). This is due to the sliding window method. The larger the window is, the longer the delay will be. Occasionally, the classifier can wrongly predict some control states, but they do not remain in time.

## 6. Conclusions

A data fusion approach based on deep neural networks is proposed for the online monitoring of working conditions corresponding to the capacity control system of the screw compressors in chillers. Data collected from sensors, which contain measurements of the main internal variables of the compressors, are considered as a time series and segmented accordingly, taking into account their dynamics. After data processing, data fusion is performed using a model based on a 1D convolutional neural network (1D CNN) so that the control states of the compressor are estimated. Once the model is built with acceptable performance, the online monitoring of the screw compressor can be undertaken.

A validation process was performed using data from an air-cooled chiller with three compressors at the Hospital of León, corresponding to two months during 2019 winter. The sensors correspond to evaporating (pressure and temperature), condensing (pressure and temperature), and current at each compressor. The control states in the compressor include Off-Ready—off but ready to start; Holding—no changes in cooling capacity; Loading—increasing cooling capacity; and Unloading—decreasing cooling capacity of the compressor.

Only data from one compressor was used for the construction of a model, and the results indicate a reliable classification of control states, i.e., with values of 0.92 for F1-score metrics for the classification of multiple classes, and all areas of hte PR curves also have values above 0.9. The model is selected considering its performance in comparison with other recent advances in data fusion from the literature, such as multilayer perceptron, multichannel deep convolutional neural network, and time Le-Net. Although time Le-Net has similar values of F1-score, 1D CNN requires less time for training and has a simpler network architecture, so it is selected. Furthermore, the model is evaluated using data from the other compressors of the chiller, with F1-score values of of 0.86 and 0.87 for compressors 1 and 2, respectively, which indicates the good versatility of the approach. Nevertheless, it is highly recommended that the model be trained using sensor data from a set of compressors.

Finally, a simple fusion method is also performed for fault diagnosis so that large differences between real control states and the results of the model indicate anomalies such as possible internal failures or malfunctions of any sensor, which should be analyzed. Thus, two possible failure scenarios of the compressor are simulated in terms of considering potential alarms. The mismatching in the predicted states and the real ones is clearly shown for both simulated cases of study, which reveals that it can be a suitable method for primary fault detection in the compressors in real time.

## Figures and Tables

**Table 1:** Main control states in screw compressors.

Number	Name	Description
0	Off-Ready	Compressor is ready, but not required by the chiller control.
1	Holding	The temperature of output water is within the control band. No changes in control capacity are required.
2	Loading	The temperature of output water is out of the control band (higher). Loading solenoids must be pulsed in order to increase the cooling capacity.
3	Unloading	The temperature of output water is out of the control band (lower). Unloading solenoids must be pulsed in order to decrease the cooling capacity.

**Table 2:** Main variables of refrigeration compressors.

Symbol	Name	Unit
Te	Evaporating temperature	${}^{\circ }$C
Pe	Evaporating pressure	KPa
Tc	Condensing temperature	${}^{\circ }$C
Pc	Condensing pressure	KPa
Amp	Compressor current draw	A

**Table 3:** Performance of 1D CNN with training and validation sets from compressor 3.

Metrics	F1-micro / F1-weighted	F1-micro / F1-weighted
	*Training (70%)*	*Validation (30%)*
**Compressor 3**	0.94/0.94	0.92/0.92

**Table 4:** Comparison of the performance with state-of-the-art methods (in bold, the best ones).

Methods (Compressor 3)	F1-micro/F1-weighted	Training Time [seconds]	Validation Time/Sample [Milliseconds]
**1D CNN**	**0.92/0.92**	**243.5**	**145.3**
MLP	0.81/0.80	1158.2	441.1
MCDCNN	0.90/0.90	717.5	391.6
TimeLeNet	0.92/0.92	354.6	237.5

**Table 5:** Performance of 1D CNN with test datasets from compressors 1 and 2.

Model: Compressor 3	F1-micro/F1-weighted
**Compressor 1** (*100% Dataset*)	0.86/0.86
**Compressor 2** (*100% Dataset*)	0.87/0.87

## Abbreviations

The following abbreviations are used in this manuscript:

FLA	Full load Amperage
1D	One-dimensional
CNN	Convolutional neural network
MLP	Multilayer perceptron
MCDCNN	Multichannel deep convolutional neural network
PR curve	Prediction–recall curve
ROC curve	Receiver operating characteristic curve
AUC	Area under the curve
IoT	Internet of Things
HVAC	Heating, ventilating, and air conditioning
EEV	Electronic expansion valve
BMS	Building management system
